# Modeling the Antioxidant Capacity of Red Wine from Different Production Years and Sources under Censoring

**DOI:** 10.1155/2013/267360

**Published:** 2013-10-24

**Authors:** Lorentz Jäntschi, Radu E. Sestraş, Sorana D. Bolboacă

**Affiliations:** ^1^Technical University of Cluj-Napoca, Department of Chemistry, 103-105 Muncii Boulevard, 400641 Cluj-Napoca, Romania; ^2^University of Agricultural Science and Veterinary Medicine Cluj-Napoca, 3-5 Calea Mănăştur, 400372 Cluj-Napoca, Romania; ^3^“Iuliu Haţieganu” University of Medicine and Pharmacy, Department of Medical Informatics and Biostatistics, 6 Louis Pasteur, 400349 Cluj-Napoca, Romania

## Abstract

The health benefit of drinking wine, expressed as capacity to defend the human organism from the free radicals action and thus reducing the oxidative stress, has already been demonstrated, and the results had been published in scientific literature. The aim of our study was to develop and assess a model able to estimate the antioxidant capacity (AC) of several samples of Romanian wines and to evaluate the AC dependency on the vintage (defined as the year in which wine was produced) and grape variety under presence of censored data. A contingency of two grape varieties from two different vineyards in Romania and five production years, with some missing experimental data, was used to conduct the analysis. The analysis showed that the antioxidant capacity of the investigated wines is linearly dependent on the vintage. Furthermore, an iterative algorithm was developed and applied to obtain the coefficients of the model and to estimate the missing experimental value. The contribution of wine source to the antioxidant capacity proved equal to 11%.

## 1. Introduction

The antioxidant capacity of food constituents and the role of antioxidants in human health found attention in the recent years [1]. The antioxidant capacity is translated by the capacity to defend an organism from the action of free radicals and consequently to prevent the disorders deriving from persistent antioxidant stress [2, 3]. Researches were carried out to identify the role of antioxidants as adjuvant treatment of different diseases such as pulmonary hypertension [4], diabetic kidney disease [5], insulin sensitivity in type 2 diabetes mellitus [6], cancer [7], periodontal diseases [8], and cardiovascular disease [9].

A series of food constituents with antioxidant capacities had been identified: tea (green tea leaves were found to have high phenolic content [10]), citrus fruits [11, 12], grape [13], apples [14, 15] and peaches [16], strawberries [7, 17], raspberries and blueberries [18], cherries [19], kiwi fruit [20, 21], plum [22], melon [23], chickpeas [24], carrots [25], peppers [26, 27], vegetable [28], and so forth. 

The antioxidant activity of wines and grapes was lately of interest for many researchers. Several antioxidant compounds such as flavanol, hydroxybenzoic acids, hydroxycinnamic acids, tartaric acid derivatives, proanthocyanidins, phenols, flavonols, anthocyanins, and resveratrols have been identified in wines and grapes [29]. Lachman et al. [29] identified the following factors that influence the antioxidant activity in grapes and wines: grape varieties and cultivars (high total polyphenols in blue grapes and less content in white varieties), vintage (the year in which wine was produced), vineyard region (location and climatic conditions), winemaking process, storage conditions, and wine age. Antioxidant activity of grapes and wine had been studied all over the world and varieties with high antioxidant capacity were identified: Pinot Noir, Egiodola, Merlot and Chardonnay varieties (France [30]), Cabernet Sauvignon (France [30], Serbia [31], Chile [32], China [33], Macedonia [34], Australia [35], Romania [36], South America [37]), Muscat (Romania [38], South Korea [39]), Syrah (France [30], Greece [40], Portugal [41], South America [37]), Malbec (South America [37]), and so forth.

The antioxidant capacity of wines produced in 1995, 2000, 2002, 2003, and 2005 in Romania had been previously determined [36]. Two grape varieties with missing data in contingency led to the following objectives of this study: (1) identify a good mathematical model able to estimate the antioxidant activity; (2) develop an iterative algorithm able to identify most probable missing values of antioxidant activity (predictive power); and (3) estimate the missing values of antioxidant activity using the identified algorithm.

## 2. Materials and Methods

Seven samples of wine selected from Cabernet Sauvignon and Merlot varieties grown in Romania (Recaş vineyards in Timiş County and Miniş vineyards in Arad County) with different years were analyzed. The antioxidant content (see Table 1) of the investigated sample of wines was taken from [36] (the analysis being done in June 2010) and was obtained with the following formula [42]:

(1) $$\begin{array}{c} \mathrm{AC}\;\left(\%\right)=\frac{\left[\left(S_{0}-S_{20}/S_{0}\right)\right]}{100}, \end{array}$$

where AC⁡  (%) = antioxidant content expressed as percentages; *S*_0_ = baseline electron spin resonance spectroscopy (EPR) signal of the free radicals; *S*_20_ = EPR signal of the free radicals after 20 minutes following adding the extracts of wines.

The experimental antioxidant content was summarized as a contingency of an ordinal variable (vintage years) and a categorical variable (variety of grapes and vineyard) (see Table 1).

It had been previously proved that the hypothesis of independence between vintage year and vineyard as factors of antioxidant content could not be rejected for {2003,2005}×{CSI, TM1} subgroup (*X* ^2^({2003,2005}×{CSI, TM1}) = 0.03; *p*_*χ*^2^_(0.03,1) = 0.86) [36].

The steps applied in our censored data analysis were as follows:

- verify if the linearity between antioxidant content and vintage (year in which the investigated wine was produced) is true for experimental data included in the analysis. A significant linearity was identified when 8 experimental data were investigated (including also the Pinot Noir from Recaş vineyard) [36].
- If linearity exists,
- verify if the linearity between antioxidant content and wine age also exists;
- use the obtained mathematical model to estimate the antioxidant content for missing data based on available experimental data.  Table 2 presents the estimated values, the experimental values and the expected values;
- estimate the missing values (using the observed data presented in Table 2) by applying the following steps:
  - obtain the coefficients {*a*,…, *f*} using regression analysis;
  - fill in the missing values with estimated values;
  - repeat the following:
    - obtaining expected values;
    - calculating *X* ^2^ using observed and expected values;
    - filling in the missing values from Table 1 with the expected values;
    - obtaining the coefficients {*a*,…, *f*} using regression analysis;
    - filling the missing values from Table 1 with estimated values;
  - till the difference between the values of *X* ^2^ for two consecutive cycles is not statistically significant.

## 3. Results and Discussion

A linear relationship between antioxidant content and vintage has been identified for investigated samples when both observed and estimated values were analyzed:

(2) AC⁡ (%)=9215(±8038)−4.58(±4.02)·Year,r=0.8,  radj2=0.56,  F-value=8.6,pF=0.03,  t(9215)=2.95,  t(4.58)=2.93,pt=2.93=0.03,  n=7,

where AC⁡  (%) = antioxidant content (%), Year = year when the wine was produced, *r* = correlation coefficient; *r*_adj_^2^ = adjusted determination coefficient; *F*-value = Fisher's statistics; *p*_*F*_ = probability associated to *F*-value; *t* = Student *t*-value associated to intercept and to coefficient; *n* = sample size.

The observed linearity is not significantly different by the one previous identified (*r* = 0.82), when 8 observations were investigated [36].

Taking into consideration that all investigated samples were analyzed in the same year (more specifically, for these samples in the same month, June 2010), the variable Year in the equation above contains a constant term (2010). Thus, a linear relationship between antioxidant content and wine age also exists and has the same statistical characteristics as the equation above:

(3) $$\begin{array}{c} \mathrm{AC}\;\left(\%\right)=9.7\left(\pm 35\right)+4.58\left(\pm 4.02\right)\cdot \text{Wine}\_\text{Age}, \end{array}$$

where Wine_Age = the age of investigated wine expressed in years old.

Considering the above linearity relationship, also the equation without the intercept is valid:

(4) AC⁡ (%)=5.61(±1.35)·Wine_Age,r=0.8,  radj2=0.43,pF=0.03,  t(5.61)=10.2,pt=5.61=5·10−5,  n=7.

However, more important than that, we are interested in ageing of the wines for each vineyard.

**Figure 1:**
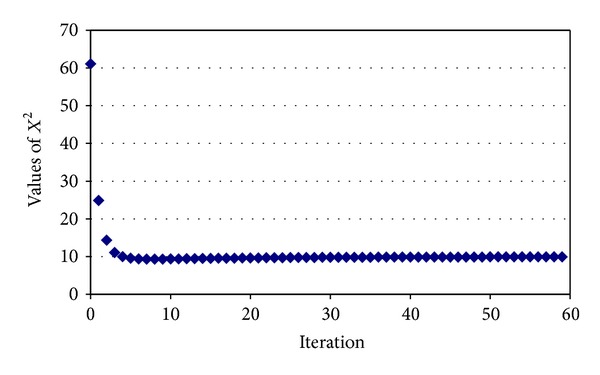
Evolution of *X* ^2^ statistics as function of iteration.

**Figure 2:**
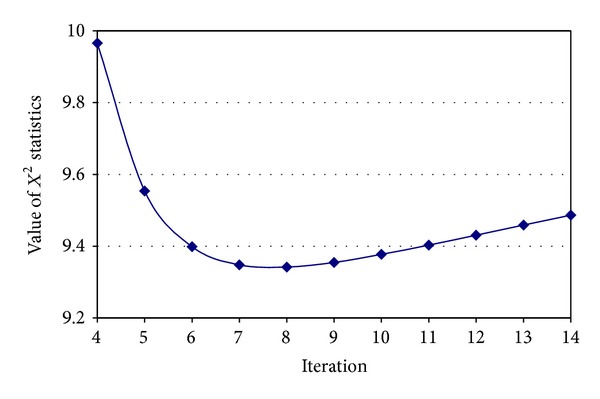
Zoom in *X* ^2^ optimization in the neighborhood of minimum.

The proposed estimation approach was applied on experimental data presented in Table 1, and the evolution of *X* ^2^ as function of iteration is presented Figure 1. The zoom at the level of which *X* ^2^ statistics cross the minimum value is detailed in Figure 2.

Analysis of Figures 1 and 2 revealed that the values of *X* ^2^ statistics did not converge to a global minimum. The local minimum is reached in the 7th iteration, and a slight increase in the values of *X* ^2^ is observed after this iteration. A difference lower than 10^−4^ between consecutive *X* ^2^ values led to the stop of the algorithm after the 59th iteration (Figure 1). The obtained estimated values were used to fill in the missing values in Table 1, and based on observed/estimated values, the expected values were calculated (Table 3).

**Figure 3:**
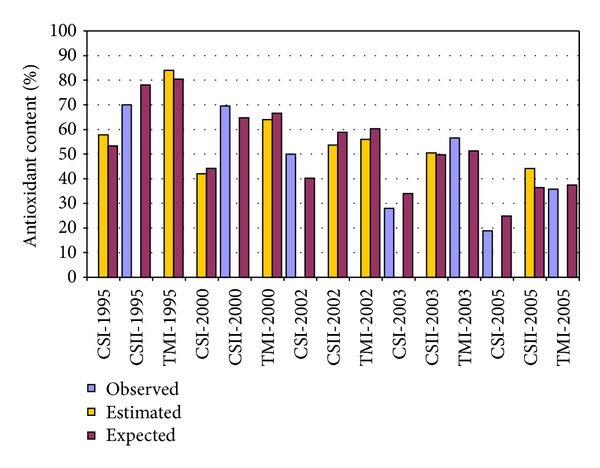
Observed, estimated, and expected antioxidant content of investigated wines (CSI = Cabernet Sauvignon from Recaş vineyard; CSII = Cabernet Sauvignon from Miniş vineyard; TMI = Merlot from Recaş vineyard).

Graphical representation presented in Figure 3 shows how well the estimated (through regression) and expected values fit the experimental values.

**Figure 4:**
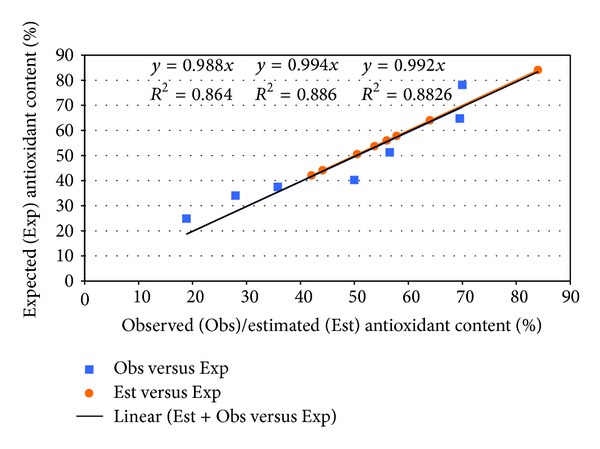
Regressions between observed (Obs), estimated (Est), and expected antioxidant content.

The regression analysis between expected and observed/estimated antioxidant content was conducted, and the results is presented in Figure 4.

The regression models obtained for different investigated wines are as follows.

(i) CSI (Cabernet Sauvignon from Recaş vineyard):
  (5) AC⁡ (%)=5444(±2889)−2.7(±1.4)·Year,AC⁡ (%)=15(±14)+2.7(±1.4)·Wine_Age,r=0.96,  radj2=0.90,  pF=0.01,AC⁡ (%)=4.2(±0.9)·Wine_Age,r=0.78,  radj2=0.36,  pF=0.09.
(ii) CSII (Cabernet Sauvignon from Miniş vineyard):
  (6) AC⁡ (%)=7967(±4228)−4.0(±2.1)·Year,AC⁡ (%)=22(±20)+4.0(±2.1)·Wine_Age,r=0.96,  radj2=0.90,  pF=0.01,AC⁡ (%)=6.1(±1.3)·Wine_Age,r=0.78,  radj2=0.36,  pF=0.09.
(iii) TMI (Merlot from Recaş vineyard):
  (7) AC⁡ (%)8204(±4353)−4.1(±2.2)·Year,AC⁡ (%)=23(±21)+4.1(±2.1)·Wine_Age,r=0.96,  radj2=0.90,  pF=0.01,AC⁡ (%)=6.3(±1.3)·Wine_Age,r=0.79,  radj2=0.37,  pF=0.08.

The analysis of identified relationships revealed the following.

- The identified relationships are not significantly different from each other at a significance level of 5% since the 95% confidence intervals of coefficients overlap each other. As a result, the conclusions regarding a significant difference could not be sustained at a risk of error equal to 5%.
- The intercept provided a measure of the antioxidant quantity that can be obtained by wine ageing. According to this criterion, the descending classification of wine in regard of antioxidant content is Merlot-Cabernet Sauvignon-Miniş (distinct from TMI at risk to be in error of 91%)-Cabernet Sauvignon-Recaş (distinct from TMI at risk to be in error of 19%, and distinct from CSII at a risk to be in error of 21%).
- The slope gives a measure of speed of ageing. Merlot aged faster, and it is closely followed by Cabernet Sauvignon-Miniş (distinct from TMI at a risk of error equal to 91%) and it is followed by Cabernet Sauvignon-Recaş (distinct from TMI at a risk of error of 19% and from CSII at a risk of error equal to 21%).
- The investigated wines come with an original richness in antioxidants since all models that assumed that the amount of antioxidants is null in the year when the wine was produced are rejected (*P* values ≥0.08). Furthermore, the antioxidant capacity is enriched annually with aging, and this enrichment is different for each brand.

Regression analysis of all data included in this study, analysis conducted using also the expected values, provided the following result:

(8) AC⁡ (%)=7205(±3500)−3.57(±1.75)·Year,AC⁡ (%)=19.9(±16.8)+3.58(±1.74)·Wine_Age,r=0.77,  radj2=0.57,AC⁡ (%)=5.5(±0.7)·Wine_Age,r=0.63,  radj2=0.33,  pF=0.01.

The above-presented equation shows that 57% of the observed variance in antioxidant content is linearly related to the wine age. Subtracting from the total variance of 200.5, the quantity explained by wine aging (114.1–57%) and by experimental error (64.6–32%) remains a variance of 11% (21.8) due to the source of the wine. Forcing the regression line through the origin obtained a significant linear model but its performances are decreased compared with the model with the intercept, and just 33% of the observed variance in antioxidant content is linearly related to the wine age leading to an invalid model. The presented results showed that our algorithm was able to provide reliable estimation of antioxidant activity on the investigated sample of wines. The identified linearity between antioxidant capacities and the wine age (obtained with 7 observations) was not a surprise because similar results had been previously identified and reported [29, 43, 44]. The reliability of the applied approach is sustained by the fitting of estimated and expected values (Figure 3), observed-expected, and estimated-expected linearity (Figure 4) as well as by the characteristics of the regression models. Wines come with an original richness in antioxidants since the models that assumed the absence of the antioxidant capacity in the year when the wine was produced, and antioxidant capacity increased annually with wine aging. The equations obtained and presented in this paper showed this. In our study, the influence of the type of flavonoids and/or nonflavonoids (according to the number of OH and OCH_3_ groups and their positions on the ring) [44], total polyphenol and total flavanol concentrations [45], possible synergy or antagonism among the different classes of polyphenols [46], and of the anthocyanin composition of red grape cultivar and their corresponding single-cultivar wine [47] is embedded in the “vineyard”.

## 4. Conclusions

Our algorithm proved to be able to operate on contingency table with gaps (censored data), and the resulting solution is not a trivial solution in relation to minimizing the *X* ^2^ statistics and thus to minimize the risk of being in error. The equations obtained for antioxidant capacity showed small differences (besides being statistically significant) in antioxidant capacity of wines from different varieties of grapes that allows obtaining an equation of antioxidant capacity as function of wine age for all samples included in the study.

## Figures and Tables

**Table 1:** Mean values of antioxidant content.

Vintage	Vineyards		
	CSI (%)	CSII (%)	TMI (%)
1995		70.01	
2000		69.54	
2002	50.00		
2003	27.98		56.56
2005	18.86		35.80

CSI: Cabernet Sauvignon from Recaş vineyard.

CSII: Cabernet Sauvignon from Miniş vineyard.

TMI: Merlot from Recaş vineyard.

**Table 2:** Experimental design for antioxidant content estimation: observed and expected contingency table.

Vintage		Source			
		CSI	CSII	TMI	*∑*
1995	Observed/estimated	*a* · 1995 + *b*	70.01	*e* · 1995 + *f*	∑_1995_
	Expected	∑_1995_ · ∑_CSI_/∑_∑_	∑_1995_ · ∑_CSII_/∑_∑_	∑_1995_ · ∑_TMI_/∑_∑_	
2000	Observed/estimated	*a* · 2000 + *b*	69.54	*e* · 2000 + *f*	∑_2000_
	Expected	∑_2000_ · ∑_CSI_/∑_∑_	∑_2000_ · ∑_CSII_/∑_∑_	∑_2000_ · ∑_TMI_/∑_∑_	
2002	Observed/estimated	50.00	*c* · 2002 + *d*	*e* · 2002 + *f*	∑_2002_
	Expected	∑_2002_ · ∑_CSI_/∑_∑_	∑_2002_ · ∑_CSII_/∑_∑_	∑_2002_ · ∑_TMI_/∑_∑_	
2003	Observed/estimated	27.98	*c* · 2003 + *d*	56.56	∑_2003_
	Expected	∑_2003_ · ∑_CSI_/∑_∑_	∑_2003_ · ∑_CSII_/∑_∑_	∑_2003_ · ∑_TMI_/∑_∑_	
2005	Observed/estimated	18.86	*c* · 2005 + *d*	35.80	∑_2005_
	Expected	∑_2005_ · ∑_CSI_/∑_∑_	∑_2005_ · ∑_CSII_/∑_∑_	∑_2005_ · ∑_TMI_/∑_∑_	
∑		∑_CSI_	∑_CSII_	∑_TMI_	∑_∑_

CSI: Cabernet Sauvignon from Recaş vineyard.

CSII: Cabernet Sauvignon from Miniş vineyard.

TMI: Merlot from Recaş vineyard.

*a*, *b*, *c*, *d*, *e*, and *f*: coefficients to be obtained based on experimental data.

**Table 3:** Antioxidant content: estimated (values in bold) or observed values and expected values.

Vintage		Source		
		CSI	CSII	TMI
1995	Observed/estimated	**57.82**	70.01	**84.04**
	Expected	53.37	78.11	80.42
2000	Observed/estimated	**42.04**	69.54	**64.02**
	Expected	44.23	64.73	66.65
2002	Observed/estimated	50.00	**53.73**	**56.01**
	Expected	40.23	58.88	60.33
2003	Observed/estimated	27.98	**50.53**	56.56
	Expected	34.02	49.79	51.26
2005	Observed/estimated	18.86	**44.13**	35.80
	Expected	24.88	36.41	37.49

CSI: Cabernet Sauvignon from Recaş vineyard.

CSII: Cabernet Sauvignon from Miniş vineyard.

TMI: Merlot from Recaş vineyard; in bold are the estimated values.
